# The implication of FLT3 amplification for FLT targeted therapeutics in solid tumors

**DOI:** 10.18632/oncotarget.13700

**Published:** 2016-11-29

**Authors:** Sung Hee Lim, Sun-Young Kim, Kyung Kim, Hyojin Jang, Soomin Ahn, Kyoung-Mee Kim, Nayoung K.D. Kim, Woongyang Park, Su Jin Lee, Seung Tae Kim, Se Hoon Park, Joon Oh Park, Young Suk Park, Se-Hoon Lee, Ho Yeong Lim, Keunchil Park, Won Ki Kang, Jeeyun Lee

**Affiliations:** ^1^ Division of Hematology-Oncology, Department of Medicine, Samsung Medical Center, Sungkyunkwan University School of Medicine, Seoul, Korea; ^2^ Department of Pathology and Translational Genomics, Sungkyunkwan University School of Medicine, Seoul, Korea; ^3^ Samsung Genome Institute, Samsung Medical Center, Seoul, Korea

**Keywords:** solid tumors, FLT3 amplification, regorafenib

## Abstract

We investigated the patients with solid cancers harboring Fms-like tyrosine kinase 3 (FLT3) amplification using targeted sequencing of tumor tissue specimen and FISH assay. Simultaneously, FLT3-amplified patient-derived cells (PDCs) were generated to evaluate the sensitivity to FLT3 inhibition. A patient with metastatic colon cancer who was previously treated with more than 3^rd^ line cytotoxic chemotherapy was found to have FLT3 amplification and then received regorafenib showing partial response. In two PDC cell lines with FLT3 amplification, FLT3 mRNA expression was increased, however, the growth of tumor cells was not significantly inhibited by either regorafenib or sorafenib which is known to block the activity FLT3. Additional drug combinations with mTOR inhibitor did not affect the cell proliferation of PDC. FLT3 amplification in solid cancers is infrequently observed using targeted genomic profile, as yet, FLT3 amplification does not seem to be an actionable target or a proper biomarker for FLT3 inhibitor sensitivity.

## INTRODUCTION

Fms-like tyrosine kinase-3 (FLT3) is a gene that encodes for a tyrosine kinase that activates pathway in proliferation and differentiation of hematopoietic stem cells. FLT3 is frequently mutated in acute myeloid leukemia, myelodysplastic syndromes, and other hematologic malignancies [1]. Oncogenic activation of FLT3 by internal tandem duplication was found in 20-25% of AML patients [2, 3] and is well known for association with poor clinical outcome [4]. In cancer cells with activating mutations in FLT3, FLT3 inhibitors or tyrosine kinase inhibitors have been shown to be effective [5, 6] and a number of small-molecule tyrosine inhibitors of FLT3 have been developed and trials are ongoing.

The role of FLT3 gene aberration in solid tumors has not yet been established. A comprehensive analysis of FLT3 aberrations showed that the majority are somatic mutations, followed by gene amplification [7]. The incidence of FLT amplification in solid tumors was reported up to 13.8 % in breast cancer [8], 5.5% in colorectal cancer [9], 1.7% in gastric cancer [10], and 0.4% in lung adenocarcinoma [11]. Recently, a refractory colorectal cancer patient with FLT3 amplification using targeted genomic profile (FoundationOne) demonstrated clinical benefit with sorafenib, a multikinase inhibitor targeting vascular endothelial growth factor receptors (VEGFR) 1 to 3, FLT3, platelet-derived growth factor receptor (PDGFR), c-Kit protein (KIT), and RET receptor tyrosine kinases [12]. Although it remains unknown that FLT3 amplification might be potentially actionable molecular alterations in certain tumor types, in the current study, we investigated patients with solid cancers majority of gastrointestinal malignancies harboring of FLT3 amplification and the clinical efficacy of FLT3 inhibition in this subpopulation. We further generated patient derived cells (PDC) from some of these patients’ tumor tissue as previously described [13, 14] and evaluated the potential anti-tumor efficacy of FLT3 inhibitors.

## RESULTS

### Patients’ characteristics

**Figure 1:**
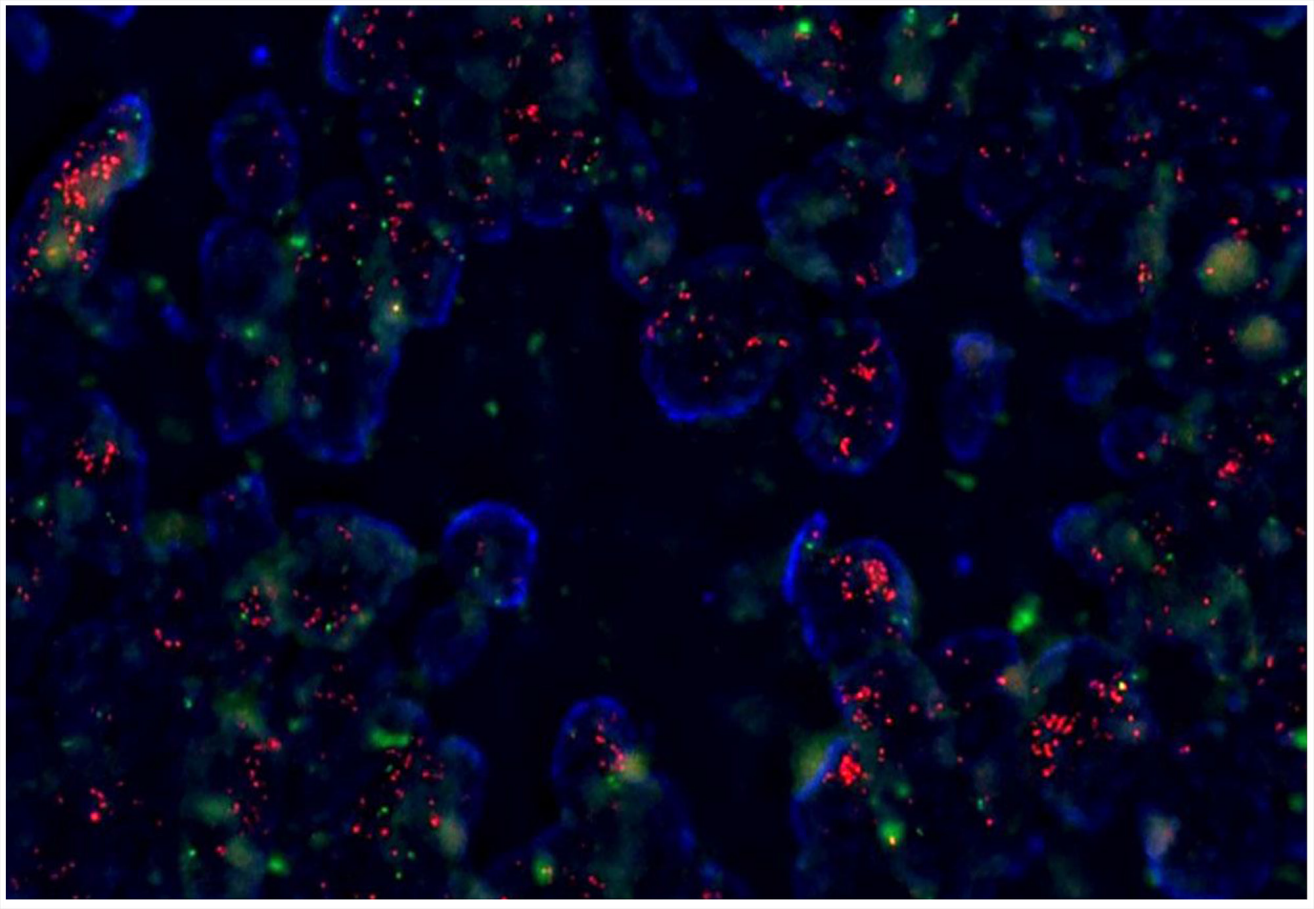
Fluorescence in situ hybridization (FISH) for FLT3 amplification

Using targeted sequencing, an Illumina HiSeq2500-based platform comprising SNV, CNV, INDEL and chromosomal rearrangements covering 381 genes, we identified twenty patients with FLT3 amplification. Table 1 and 2 provide baseline patient characteristics. The most frequent cancer types were colon cancer (n=8, 40%), followed by rectal cancer (n=7, 35%), gastric cancer (n=3, 15%), non-small cell lung cancer (n=1, 5%), and hepatocellular carcinoma (n=1, 5%). The median age of all patients was 63 years (range, 40 – 83) and 60% were male. Among gastric and colorectal cancer patients, most of the patients (83.3%) were mild to moderate differentiated and 6 (33.3%, colon cancer) had concomitant KRAS mutation. In total, 9 patients (45%) were found to have FLT3 amplification by FISH assay (Figure 1). Clinical characteristics of the 9 patients are listed in Table 3.

**Table 1:** Characteristics of FLT3 amplification patients (N=20) according to tumor types

	Colon	Rectum	Gastric	Lung	**HCC**
N	8	7	3	1	1
Age					
Median	63.5	57.0	64.0		
Range	61 - 75	49 - 64	40 - 72		
Gender					
Male	5	3	2	1	1
Female	3	4	1		
Smoking history					
Never	NC	NC	NC		NC
Ever					
Histology					
Adenocarcinoma	8	7	3		
Squamous cell				1	
Hepatocellular carcinoma					1
Tumor differentiation					
Well	2	1			
Moderate	6	5	1		
Poor	0	1	2		
Concomitant genetic aberrations					
KRAS	2	4			
BRAF					
EGFR					

**Table 2:** Patient characteristics

Variable	FLT3 amplification (+) by targeted sequencing
	N=20
Age	
Median	63.5
Range	40 – 83
Gender, no (%)	
Male	8 (40)
Female	12 (60)
Tumor type, no (%)	
Gastric cancer	3 (15)
NSCLC	1 (5)
Colon cancer	8 (40)
Rectal cancer	7 (35)
Hepatocellular carcinoma	1 (5)
FLT amplification by FISH (%)	9 (45)

**Table 3:** Clinical characteristics of the 9 patients with FLT3 amplified tumors by FISH

Case ID	Gender	Age	Site of tumor	Histology	Stage	Copy number	FLT3/CEP13	Dead/Alive	Treatment	Best response
PS_CS_15_201	F	53	Rectum	adeno	IV	5-10	4:1	Alive	Regorafenib	SD
PS_CS_15_469_N	F	62	Colon	adeno	IV	15-30	10:1	Alive	Regorafenib	PR
PS_CS_15_039	F	57	Rectum	adeno	IV	6-12	5:1	Dead	Regorafenib	PD

### Case

**Figure 2:**
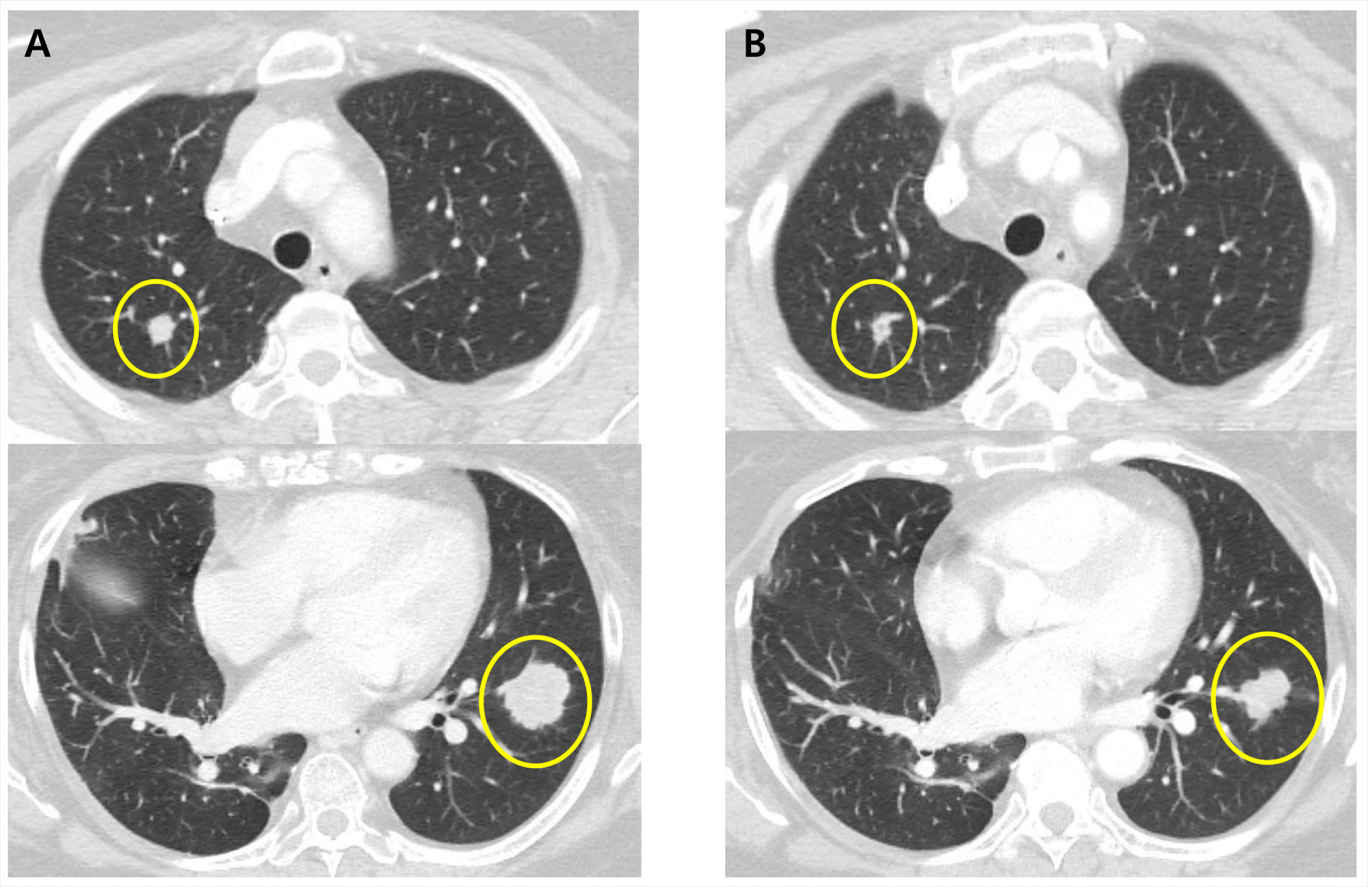
CT scans of the chest in patient with metastatic colorectal cancer harboring FLT3 amplification **A**. CT of the chest showing multiple metastatic nodules in the both lung before regorafenib **B**. CT of the chest showing response to treatment with regorafenib.

A 62-year-old female patient who presented with sigmoid colon cancer with lung metastasis had recurrent diseases in lung after curative resection followed by 8 cycles of adjuvant XELOX (capecitabine and oxaliplatin) chemotherapy. Molecular studies revealed the presence of KRAS G12S mutation in the primary colon cancer tissue specimen. The patient recurred with multiple lung metastases 4 months after completion of adjuvant FOLFOX chemotherapy. She started palliative chemotherapy with avastin/FOLFIRI (irinotecan, leucovorin and fluorouracil) for eight cycles but progressed. Then the patient has been treated with regorafenib and achieved partial response (Figure 2). Copy number of FLT3 amplification was 15-30 in FISH assay and the FLT3/CEP13 ratio was 10. In this particular patient, patient was on regorafenib for 12.4 months with partial response.

### Patient derived tumor cells

**Figure 3:**
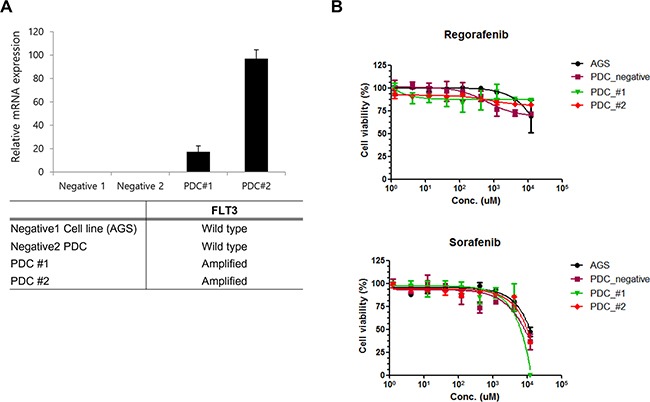
**A**. qPCR validation of FLT3 amplification in PDC. **B**. Cell proliferation inhibition curve of regorafenib and sorafenib on FLT3 amplified PDC.

**Figure 4:**
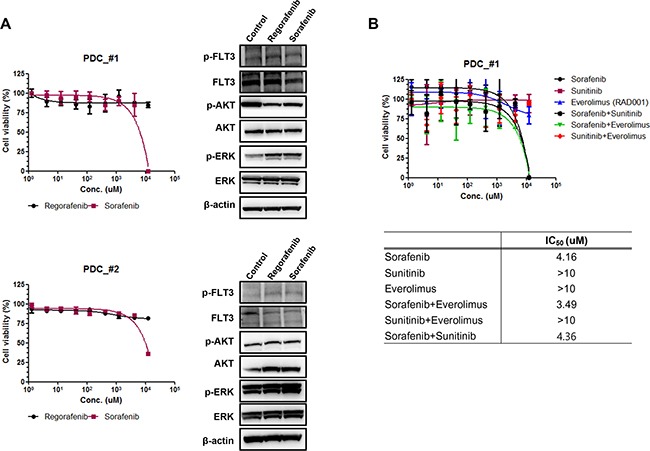
**A**. The expression of downstream signaling molecules after treatment of regorafenib and sorafenib on FLT3 amplified PDC#1 and #2. **B**. Cell proliferation inhibition curve of combination drug treatment to the PDC#1; sorafenib, sunitinib, everolimus, sorafenib+everolimus, sunitinib+everolimus, and sorafenib+sunitinib.

In order to investigate the sensitivity of patient derived tumor cells on regorafenib and sorafenib, we generated malignant tumor cells from FLT3 amplified tissues as described in Methods. First we measured relative mRNA expression of FLT3 via qRT-PCR. As shown in Figure 3A, FLT3 expression was increased in PDC#1 and PDC #2 compare to negative cell line AGS and PDC. PDC#1 and PDC#2 were tested the anti-tumor effect of regorafenib and sorafenib, the growth of tumor cells was not considerably reduced by exposure of inhibitors compared with control *in vitro* cell viability assay (Figure 3B). We further detected FLT3 expression and regulation of downstream targets upon exposure to each drug via immunoblot assay. We observed that FLT3 expression was slightly reduced by treatment of regorafenib or sorafenib, but downstream targets such as phospho-AKT or phospho ERK were not affected by exposure of each drug (Figure 4A). We next tested combination cell viability assay with additional drug treatment to the PDC#1; sorafenib, sunitinib, everolimus, sorafenib+everolimus, sunitinib+everolimus, and sorafenib+sunitinib. Cell proliferation of PDC was not significantly inhibited by these combinations (Figure 4B).

## DISCUSSION

FLT3 alterations are well documented in leukemia and therapeutic compounds are being developed to inhibit the activity of the constitutively active FLT3 receptors [15, 16]. In this study, the incidence of FLT3 amplification is 1.6% (20 patients) and the majority was gastrointestinal tumors (colorectal and gastric cancers). In the TCGA data set, the incidence of FLT3 amplification was highest in colorectal cancer (34.6%), breast cancer (10.5%), and gastric cancer (8.3%) in the order of frequency in all FLT3-amplified cancer (Supplementary Figure S1). We found a previously heavily treated patient with metastatic colon cancer harboring FLT3 amplification by targeted sequencing and the patient showed partial response to regorafenib for 12.4 months. Based on this, we hypothesized that FLT3 amplification might be a potential therapeutic target in colorectal cancer and investigated further using FLT3-amplified PDC from colorectal cancer patients. In two PDC cell lines with FLT3 amplification, with increased FLT3 mRNA expression, tumor growths were not significantly inhibited by either regorafenib or sorafenib. Regorafenib and sorafenib are multikinase inhibitors that block the activity of multiple protein kinases, mainly tumor angiogenesis (VEGFR1-3) and also including RET, FLT3, KIT, PDGFR, FGFR1-2, TIE2, RAF-1, BRAF, BRAFV600E and Abl pathways.

FLT3 amplification has been previously detected in approximately 3% of CRC samples [17, 18]. It was reported to be associated with primary or acquired resistance to EGFR blockade in colorectal cancers using circulating tumor DNA (ctDNA) [19]. Recently, Braxton et al. reported that FLT3 amplified cases display a high number of pathogenic variant when compared to FLT3 wild-type cases, suggesting that FLT3 amplification in mismatch repair proficient colorectal adenocarcinoma occurred as a late event [20]. However, they suggested that FLT3 amplification in CRC may not be the most effective genomic alteration for therapy and seems to be a passenger alteration [20]. Among 50 gastric cancer tissues, FLT3 gene amplification (3.7-fold) was noted only in one sample. And the MAPK pathway was activated by mutations and gene amplifications of ERBB2, FLT3, and KRAS [21].

So far, the FLT3 signaling cascade has not been definitively characterized, complex associations and downstream effects that probably occur after activation of FLT3. Binding of FLT3 ligand to FLT3 triggers the PI3K (phosphatidylinositol 3-kinase) and RAS pathways, leading to increased cell proliferation and the inhibition of apoptosis [4]. Therefore, FLT3-amplification may not be the proper biomarker for FLT3 inhibitor sensitivity, and molecular mechanisms in addition to FLT3 amplification may be involved in the oncogenesis of FLT3-amplified solid cancers. Even in patients with FLT3-mutated leukemia, a number of small molecule tyrosine kinase inhibitors with activity against FLT3 have been developed [22, 23] but, as of yet, none of the studied FLT3 inhibitors has not been used for routine clinical use in FLT3-mutated AML. This is in part due to the insufficient antitumor effect observed with most FLT3 inhibitors alone and rapid development of resistance which may illustrate the need for additional therapeutics [24].

In summary, FLT3 amplification in solid cancers is a frequently encountered genomic alteration in the clinic with various prevalence across cancer types. However, based on our data, FLT3 may not be an “actionable” target, at least in colorectal cancer, either as monotherapy or combined therapy. We are currently investigating other genomic alterations that may be associated with regorafenib in the patient case presented in this paper.

## MATERIALS AND METHODS

### Patients

This investigation was conducted in accordance with the ethical standards of the Declaration of Helsinki and national and international guidelines, and was approved by the Institutional Review Board at Samsung Medical Center. Between October 2013 and Jan 2016, 1,250 patients with gastrointestinal cancer, rare cancer, and lung cancer were prospectively enrolled in the screening program at Samsung Medical Center, Seoul, Korea using targeted sequencing. Patient inclusion criteria were as follows; age ≥18 years, pathologically confirmed cancer, resection/ biopsies of the primary or metastatic site, and available data on clinicopathologic characteristics.

### Targeted exome sequencing

Genomic DNA was extracted, and a SureSelect customized kit (Agilent Technologies, Santa Clara, CA, USA) was used for capturing 83 or 379 cancer-related genes depending on the version of sequencing panel. Illumina HiSeq 2500 was used for sequencing with 100 bp paired-end reads. The sequencing reads were aligned to the human genome reference sequence (hg19) using BWA-mem (v0.7.5), SAMTOOLS (v0.1.18), Picard (v1.93), and GATK (v3.1.1) for sorting SAM/BAM files, duplicate marking, and local realignment, respectively. Local realignment and base recalibration were carried out based on dbSNP137, Mills indels, HapMap, and Omni. SNVs and InDels were identified using Mutect (v1.1.4) and Pindel (v0.2.4), respectively. ANNOVAR was used to annotate the detected variants. Only variants with over 1% of allele frequency were included in the results. Copy number variations were calculated for targeted sequencing regions by dividing read-depth per exon by the normal reads per exon using an in-house reference. Translocations in the target region were identified using an in-house algorithm (*in preparation*).

### FLT amplification by fluorescence in situ hybridization (FISH) assay

Tumor sections were cut to 1 μm thickness, followed by deparaffinization with the pretreatment reagent (Abbott, 30-801250) at 80°C for 30 min. Protease digestion procedures were performed using the protease reagent (Abbott, 30-801255) at 37°C for 20 min. *FLT3* probes (Empire genomics, FLT3-20-RE) and Chr 13 control probe (Empire genomics, CHR13-10-GR) were hybridized at 73°C for 5 min and 37°C for 20 h. After hybridization, the slides were washed in 2× saline-sodium citrate/0.3% NP-40 at 72°C for 5 min, air dried, and counterstained with 4′, 6-diamidino-2-phenylindole (DAPI) I and DAPI II (Abbott Molecular). The slides were examined under a fluorescence microscope equipped with Spectrum Texas Red with isothiocyanate and DAPI filters. The *FLT3*/Chr13 ratio was established after counting at least 50 tumor cell nuclei. An *FLT3*/Chr13 ratio higher than 2.0 was interpreted as gene amplification-positive.

### Patient-derived cell culture

Tumor tissue from patients with metastatic cancer was removed surgically or biopsy from patients who had provided informed consent. Collected tissue was minced and dissociated with enzymatic method. The cells were grown in RPMI 1640 supplemented with 10% fetal bovine serum (FBS; Gibco BRL, Paisley, UK) and 1% antibiotic-anti-mycotic solution (Gibco BRL), 0.5 μg/ ml of hydrocortisone (Sigma Aldrich), 5 μg/ ml of insulin (PeproTech, Rocky Hill, NJ, USA), 5 ng of EGF and 2.5 ng of FGF (PeproTech). The medium was changed every 3 days, and cells were maintained at 37°C in a humidified 5% CO2 incubator. PDCs were passaged using TrypLE Express (Gibco BRL) to detach cells when the cells reached 80–90% confluence.

### Quantitative real-time reverse transcription-polymerase chain reaction

Total RNA was isolated from cells using the Qiagen RNeasy Mini Kit protocol (Qiagen, Valencia, CA, USA) according to the manufacturers’ recommendations. Total RNA from each sample was reverse transcribed with the SuperScript®III First-Strand Synthesis System using Oligo(dT) 20 primer (Invitrogen Corp, Carlsbad, California, USA). All qPCR reactions were performed with a 7500 Fast Real-Time PCR System (Applied Biosystems, Foster City, CA), using FLT3 primer Forward:5’-CCATCTTGGACCTGGAAGAA-3’ Reverse:5’-AAGATGTGCCAAGGGAATTG-3’, and GAPDH (Forward:5’-ACA ACT TTG GTA TCG TGG G-3’ Reverse:5’- GCC ATC ACG CCA CAG TTT C-3’) was used as the housekeeping gene for relative quantification. All genes were run in triplicate to allow for the assessment of technical variability.

### Cell treatment and viability assay

After pathologic confirmation, cells were seeded on 1~2 × 10^6^ cells/10 mm dishes or 5000 cells/ well/ 96well plate for analysis of immunoblotting and cell proliferation inhibition assay and treated for 3~5 days with various doses of drugs, as indicated. Cell proliferation inhibition was determined via Cell Titer Glo (Promega, Madison, WI, USA) according to the manufacturer's protocol.

### Immunoblot analysis

Total proteins from PDCs were isolated using RIPA buffer (Sigma-Aldrich, St. Louis, MO, USA) containing a protease inhibitor cocktail (Roche, Mannheim, Germany) and phosphatase inhibitor cocktail (Roche), and protein concentrations were determined according to Bradford procedure using a Quick Start Bradford Protein Assay (Bio-Rad, Hercules, CA, USA). Thirty μg of proteins were subjected to 10 % SDS-polyacrylamide gel electrophoresis, and electro-transferred onto nitocellulose membranes. The membranes were blocked with 5 % nonfat dry milk in Tris-buffered saline containing 0.1% v/v Tween 20, and probed overnight at 4°C with a Specific antibodies: phospho-FLT3(Tyr591) and FLT3(8F2), pAkt (Ser473), Akt(C67E7), pERK1/2 (Thr202/Tyr204), ERK1/2 (Thr202/Tyr204) from Cell Signaling Technology (Beverly, MA, USA), and beta actin from Sigma Aldrich. Horseradish peroxidase-conjugated anti-rabbit or mouse IgG (Vector, Burlingame, CA, USA) were used as a secondary antibody, and signals were detected by chemiluminescence using ECL Western Blotting Substrate (Thermo Scientific, Rockford, IL, USA), and visualized by using LAS-4000 (Fujifilm, Tokyo, Japan).

### Statistical method

For PDC viability curves, results are expressed as the means. Paired one way ANOVA tests were used to calculate the P values. Statistical significance was assessed using one way ANOVA tests and described in the figure.

## SUPPLEMENTARY MATERIALS FIGURES


