# Supplementary material for: The implication of FLT3 amplification for FLT targeted therapeutics in solid tumors
Source: Oncotarget. 2016 Nov 29;8(2):3237–45. doi: 10.18632/oncotarget.13700 (PMC5356878; doi:10.18632/oncotarget.13700)
Supplement: Supplementary file 1 [file oncotarget-08-3237-s001.pdf]

The implication of FLT3 amplification for FLT targeted therapeutics in solid tumors

SUPPLEMENTARY FIGURE

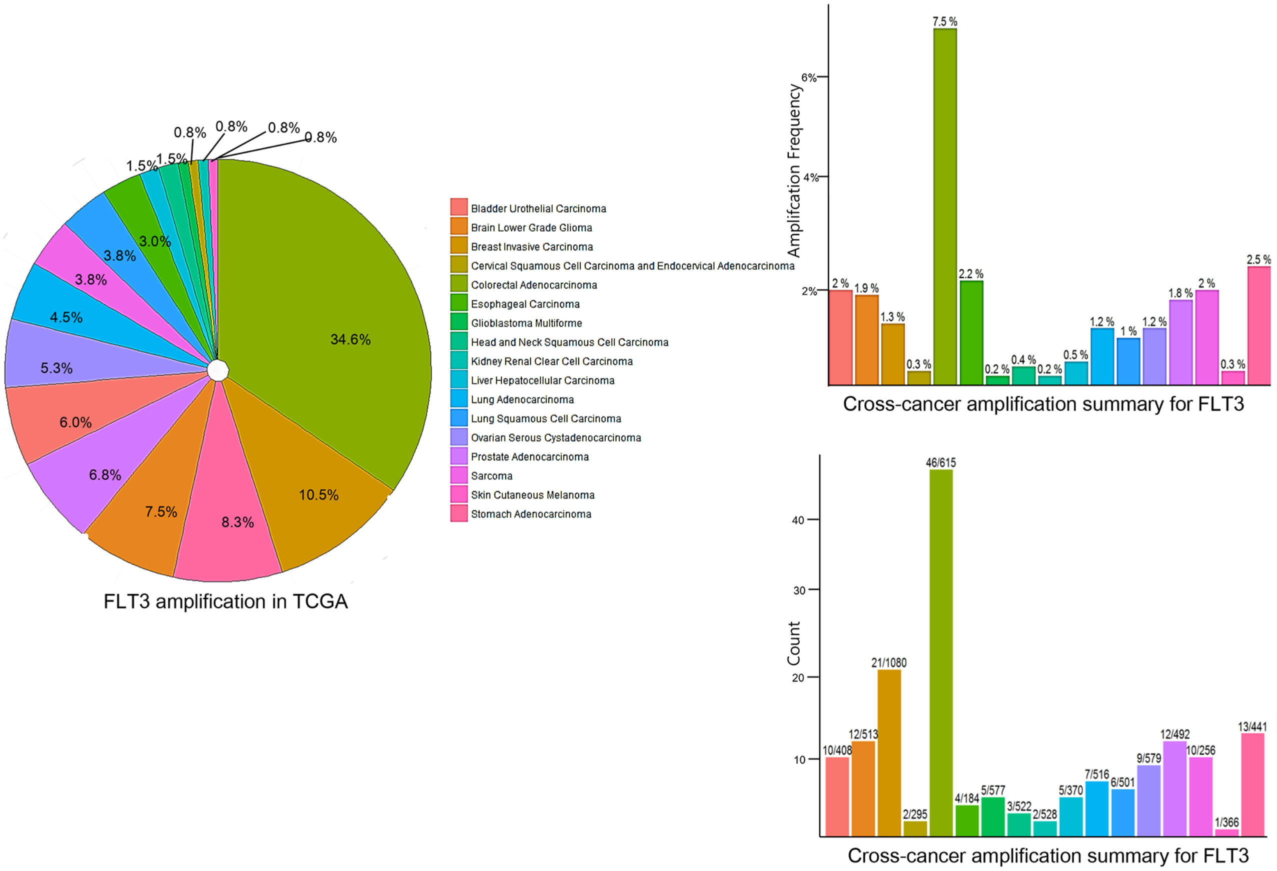

Supplementary Figure S1: The incidence of FLT3 amplification in the TCGA data set.
